# Health Literacy and Clinical Trial Participation in French Cancer Patients: A National Survey

**DOI:** 10.3390/curroncol29050253

**Published:** 2022-04-28

**Authors:** Youssoufa M. Ousseine, Anne-Déborah Bouhnik, Julien Mancini

**Affiliations:** 1 INSERM, IRD, SESSTIM, Sciences Economiques & Sociales de la Santé & Traitement de l’Information Médicale, Equipe CANBIOS Labellisée Ligue Contre le Cancer, Aix Marseille University, 13009 Marseille, France; youssoufa.ousseine@santepubliquefrance.fr (Y.M.O.); anne-deborah.bouhnik@inserm.fr (A.-D.B.); 2 Santé Publique France, French National Public Health Agency, CEDEX, 94415 Saint-Maurice, France; 3 APHM, INSERM, IRD, SESSTIM, Sciences Economiques & Sociales de la Santé & Traitement de l’Information Médicale, Equipe CANBIOS Labellisée Ligue Contre le Cancer, Hop Timone, BioSTIC, Biostatistique et Technologies de l’Information et de la Communication, Aix Marseille University, 13005 Marseille, France

**Keywords:** cancer, clinical trial participation, health literacy, VICAN survey, France

## Abstract

**Simple Summary:**

Inadequate health literacy (HL) can impair many aspects of communication, including the invitation to participate in clinical research. This study aimed to assess the relationship between HL and trial participation in French cancer patients. Our results showed that 37.6% were classified as having limited HL. One in ten (10.3%) respondents reported having been previously invited to participate in a clinical trial. Of these, 75.5% had enrolled. Limited HL was associated with fewer trial invitations but not with enrollment once invited. Addressing HL is necessary to create a more inclusive health system and to reduce inequalities not only in access to innovative cancer care, but to health inequalities in general.

**Abstract:**

Few studies have explored the relationship between health literacy (HL) and trial participation. In this context, we aimed to study this relationship in French cancer patients. We used data from the French national VIe après le CANcer (VICAN) survey. Two questionnaire items focused on previous invitations to participate in clinical trials and subsequent enrollment. The Single Item Literacy Screener was used to measure functional HL. In total, 1954 cancer patients responded to both VICAN surveys (two and five years after diagnosis). Mean age was 54.1 ± 12.7 years at diagnosis, and 37.6% were classified as having limited HL. One in ten (10.3%) respondents reported having been previously invited to participate in a clinical trial. Of these, 75.5% had enrolled. Limited HL was associated with fewer trial invitations but not with enrollment once invited. Multivariate analysis confirmed the negative effect of limited HL on clinical trial invitation (adjOR = 0.55 (0.39 to 0.77), *p* < 0.001) after adjustment for multiple characteristics. Patients with limited HL received fewer invitations to participate in trials but were likely to enroll when asked. Addressing HL is necessary to create a more inclusive health system and to reduce inequalities not only in access to innovative cancer care, but to health inequalities in general.

## 1. Introduction

Promoting participation in clinical trials is essential to ensure continuous advances in cancer care. Inviting patients to participate is often linked to a lack of or failed standard therapeutic. Given the uncertain efficacy of experimental treatments, even in the late stage of phase III trials, participation is not always associated with better clinical outcomes [1]. Nevertheless, inviting people to participate can be considered a standard of care because it usually ensures that patients will receive a promising new treatment. More generally, patient participation in research can have other positive effects. For example, it may be psychologically rewarding [2] or it may increase satisfaction with care through consent and data collection processes [3].

Given these potential benefits of clinical trials and the need for research findings that can be generalized [4], not inviting a patient to participate may be considered inequity [5]. Most of the research conducted thus far on inequity in clinical trial participation has focused on non-modifiable factors [6] such as age, ethnicity, or socioeconomic indicators [7,8,9,10,11] rather than modifiable factors such as knowledge and attitudes towards clinical trials and health literacy (HL) [12,13,14]. Understanding the term clinical trial and the vocabulary associated with trials is important for patient engagement. Previous studies have shown that many patients do not understand what a clinical trial is, and this is a barrier to participating in clinical trials [13,14]. Misunderstanding could be related to poor promotion of the information, but it is also strongly related to a low HL. HL is defined as “people’s knowledge, motivation and competences to access, understand, appraise, and apply health information in order to make judgments and take decisions in everyday life concerning healthcare, disease prevention and health promotion to maintain or improve quality of life during the life course” [15]. To the best of our knowledge, the impact of a patient’s HL on the decision to invite him/her to participate in clinical trials has not been specifically studied, despite the fact that inequities resulting from low HL are dynamic and can be successfully tackled. More specifically, although low HL has been conceptualized as a risk factor for health, HL itself has been conceptualized as a personal asset that can be developed. This can be seen in the great number of HL-improvement programs targeted at adults [16]. Moreover, age and other psychological factors are determinants, which may influence physicians’ decisions to invite patients to participate. For example, physicians might be reluctant to suggest participation to older patients, fearing refusal or agreement despite not understanding the true implications of participation, or on the contrary, that too much time would be needed to obtain consent.

Besides the inequity in the invitation to participate because of low HL, it is also important to better understand why patients with low HL who are invited to participate in clinical trials decide not to do so. The decision not to participate might be linked to one or more dimensions of HL, including the acquisition, understanding, and use of information about clinical trials [17]. Several confounding factors may also play a role in clinical trial participation (i.e., invitation or decision to participate). Low HL patients are, on average, older, reside more often in rural areas, and are more likely to have economic difficulties [18,19]. These factors may prevent them from frequenting a comprehensive cancer center [20]. Furthermore, other clinical determinants (tumor type or stage) related to a lack of prevention and insufficient screening [21] may influence clinical trial participation.

In order to better understand potential inequities in clinical trial invitation and participation in cancer patients, as well as the factors associated with these inequities, we studied the role of HL in a sample of French cancer patients participating in the VICAN survey, while accounting for age, economic hardship, cancer type, care center, and area of residency. We hypothesized that low HL was associated with not being invited to participate in clinical trials.

## 2. Methods

### 2.1. Study Design and Participants

VICAN (*VIe après le CANcer, or Life after Cancer*) is a French national longitudinal survey studying patients’ living conditions, psychosocial outcomes, and health conditions two years (VICAN2) and five years (VICAN5) after cancer diagnosis [22]. All French-speaking patients, living in France for at least two years, and diagnosed with a first malignant cancer between January and December 2011 were eligible.

The target population included individuals aged 18–82 years at the time of cancer diagnosis. Twelve cancer sites (Table 1) accounting for 88% of cancer incidence in France were included. Patients were registered in the Long Duration Disease File (*Affection Longue Durée (ALD)*) of the three main French Health Insurance Schemes, which cover over 90% of the population. The costs for diseases listed in the ALD file are fully reimbursed by the state.

**Figure 1:**
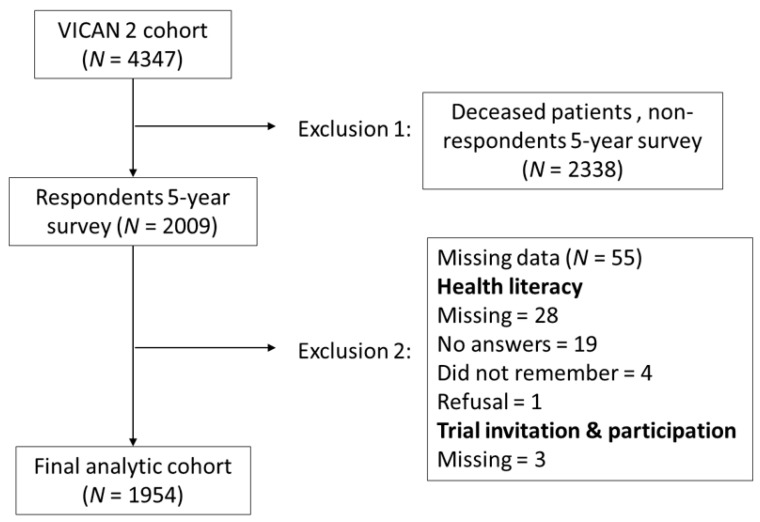
Flowchart of participants.

A stratified random sample of eligible patients was invited to participate in a computer-assisted telephone interview. A postal questionnaire was also proposed to patients with lung or upper aero-digestive tract cancers, to account for possible difficulties in responding orally. A medical survey was completed by the health care provider (HCP) who initiated the patient’s cancer treatment. Other medical information was collected from the national medico-administrative databases. The first wave of data collection took place in 2012, two years after diagnosis (VICAN2), and included 4347 cancer survivors. The second wave took place in 2015 and 2016, five years after diagnosis (VICAN5), and included 4174 individuals, 2009 of whom had already participated in VICAN2. The analyses presented here only concern the latter group (Figure 1).

For theses analyses, a weighting procedure was applied to obtain a representative sample in terms of age, gender, socioeconomic condition, cancer site, and tumor progression since diagnosis [22].

### 2.2. Variables

#### 2.2.1. Clinical Trial Invitation and Participation (Main Outcomes)

Previous clinical trial invitations and participation were self-reported in VICAN2 using the following two questions taken from an earlier cancer patient survey [11]: “Have you ever been asked to participate in a clinical trial?”, and for those who answered “yes”, “Did you agree to participate?” As participation in clinical trials was expected to mainly occur in the first years after diagnosis in this group of survivors, these two questions were asked only in the VICAN2 survey to limit recall bias. A definition of a clinical trial was provided alongside the first question (Appendix A), in order to ensure that respondents did not refer to observational research, especially given that biobank consent forms are widely available in comprehensive cancer centers [23].

#### 2.2.2. Independent Variables

Exposure variable: Functional HL was evaluated by the single item literacy screener (SILS) question: “How often do you have someone help you read hospital materials?” The SILS has proven its ability to predict functional HL evaluated using other self-reported measures [24] and objectively using the short version of the Test of Functional Health Literacy in Adults (S-TOFHLA) [25]. In line with a previous HL study [26], patients were considered to have low HL if they responded “rarely”, “sometimes”, “often” or “always” to the SILS question. HL was then divided into two categories: limited HL (“rarely”, “sometimes”, “often” or “always”) and adequate HL (“never”).

Sociodemographic characteristics: Age at diagnosis, gender, education level, cancer management center and area of residence (rural/small town/city vs. large town/city) were collected. The simplified deprivation index of the area of residence (IDS) was also determined using the town/city of residence (or ZIP code for larger towns/cities). It was developed as the first component of a principal component analysis of four socioeconomic variables (share of laborers in the active population aged 15 to 64 years, share of unemployed in the active population aged 15 to 64 years, share of graduates with a bachelor’s degree (minimum) in the population aged 15 or more not attending school and median household tax income) [27]. The IDS was divided into three categories of residence according to the quartiles (Q) in the sample, with higher values indicating higher deprivation (low deprivation: IDS < 1st quartile (Q1), high deprivation IDS > 3rd quartile (Q3), and medium deprivation Q1 ≤ IDS ≤ Q3). For example, survivors with low deprivation lived in areas with fewer laborers, less unemployment, many graduates, and high household incomes.

Financial resources were collected using the income per consumption unit (ICU). The ICU corresponds to total income (here, self-reported income) of the household divided by the number of consumption units (CU) in the household. This makes it possible to establish the disposable income per individual within the household. The number of CU is calculated from the OECD scale by summing the different individuals in the household using a different weight according to age: 1 for the first adult in the household (here, the respondent), 0.5 for other persons aged 14 years and over, and 0.3 for children under 14 years. The ICU was divided into three categories according to the quartiles in the sample, with higher values indicating greater financial resources (<Q1, Q1–Q3, >Q3).

Medical characteristics: Cancer type and metastatic status at the time of diagnosis were collected. Data from patient questionnaires, medical surveys completed by physicians, and from medico-administrative databases were combined [22] to define an individual chronic comorbidity score. This comorbidity score was constructed from the drugs reimbursed for 22 chronic diseases [28]. It was calculated as a weighted average of the chronic diseases identified for a cancer patient over a one-year period. Diseases with a high probability of hospitalization or death had the highest weights.

### 2.3. Statistical Methods

After univariate analyses using usual statistical tests (χ² test for categorical variables and ANOVA or Student’s *t* test for continuous variables), a multivariate binary logistic regression was used to model the probability of having been invited to participate in a clinical trial.

All variables with a *p* value < 0.20 in univariate analyses were eligible for the multivariate model. Age, type of residence area, cancer site and comorbidities were all systematically kept in the model. The first three factors can lead to disparity in access to clinical trials [29], while comorbidities are often associated with invitations to participate in trials [30]. The final multivariate model was built using a backward approach based on the log likelihood ratio test. Analyses were performed using SPSS 20.0 (IBM). All tests were two-sided, and the statistical significance threshold was *p* < 0.05.

## 3. Results

Of the 2009 individuals who responded to both VICAN surveys, 1954 (97.3%) responded to the SILS question and to the two questions about clinical trial invitation and participation (Figure 1). Non-respondents’ characteristics are described in a previous study [31].

The main patient characteristics are displayed in Table 1.

**Figure 2:**
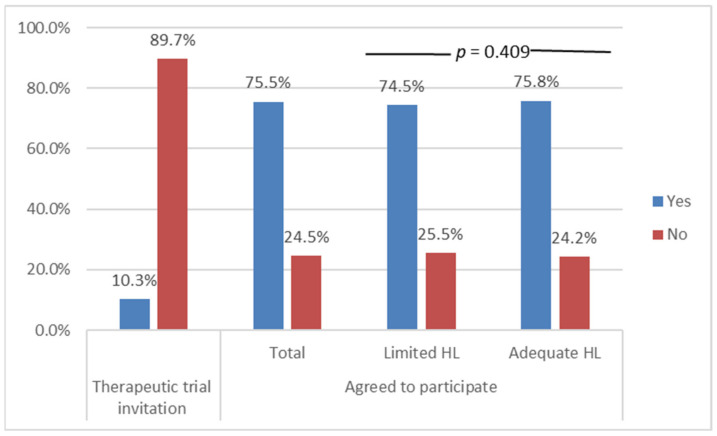
Clinical trial invitation rate and participation according to health literacy (HL) level.

Over one-third of patients (37.6%) were classified as having limited HL. One in ten patients reported that they had been invited to participate in a clinical trial. Among them, 74.5% had agreed to participate (7.7% of the total sample). One of five patients (22.8%) were over 65 years old. This group was less likely to have been invited to participate (12.5% vs. 23.9% for persons <65 years old, *p* < 0.001). However, most of those who were invited (88%) agreed to participate (5.6% of the over 65 years old). Women were significantly more likely to have been invited to participate than men (12.3% vs. 6.5% in men, *p* < 0.001). This figure was no longer significant (9.7% vs. 8.5%, *p* = 0.335) after excluding the four gender-specific cancers surveyed (breast, cervix, endometrium, and prostate). Invitations to participate in clinical trials were more likely to occur in comprehensive cancer centers (26.2%) than other management centers (private centers 7.2%, public academic centers or community centers 10.5%, *p* < 0.001). The invitation to participate did not differ according to education level. However, participants with adequate HL were more likely to have been invited to participate than those with limited HL (12.2% vs. 7.1%, *p* < 0.001). Patients with adequate HL and those with limited HL had similar agreement rates after being invited to participate (75.8% vs. 74.5%, *p* = 0.409) (Figure 2).

Multivariate analysis confirmed the negative effect of limited HL on the invitation to participate in a clinical trial (Table 2).

In multivariate analysis, other sociodemographic factors associated with not being invited were older age, having fewer financial resources, and not living in a large town/city. As for univariate analyses, education level and comorbidities were not associated with the invitation to participate. Finally, being treated in a comprehensive cancer center and for lung cancer or non-Hodgkin lymphoma were associated with a higher likelihood of being invited to participate in a clinical trial.

## 4. Discussion

To the best of our knowledge, this is the first national French study to examine the relationship between HL and both the invitation to participate in a clinical trial and patients’ agreement to do so. Limited HL was not uncommon in the cancer patients surveyed, and it was associated with a substantially lower likelihood of being invited to participate. After adjusting for multiple confounders and potential markers of ineligibility, the effect remained in multivariate analysis. This suggests that physicians might avoid proposing trial participation to patients who they believe are more difficult to adequately inform. Most patients in our sample who had been invited to take part in a clinical trial reported that they agreed to participate. No difference in agreement was observed according to HL level.

Interest in HL is recent in France, and the only reliable available estimate of its prevalence showed that 44% of the general population have limited HL [32]. It is therefore not surprising that 37% of cancer survivors in our study had limited HL. The estimated percentage of limited HL for French cancer survivors that we found is also consistent with limited HL rates observed for various states in an American study validating the SILS in patients with pathologies different from cancer (40% in hemodialysis patients in North Carolina [25] and 37.8% in diabetic patients in Ohio [33]). Furthermore, the rate of participation in our study was consistent with cancer trial participation rates reported in France.

Only one in ten patients (10.3%) in the present study reported that they had been invited to participate in a clinical trial. Of those invited, most (75.5%) agreed. This low overall participation rate (7.7%) is consistent with the rates estimated by the French national cancer institute (INCa) for 2010 (between 7.5% and 8.5%, or 11% when the estimate was based only on the incidence rate) [34].

Consistent with previous research in the United States of America and in other European countries [7], our results highlighted several inequities related to socioeconomic status and age. Patients with fewer financial resources were less likely to be invited to participate in clinical trials after adjustment for all the other characteristics. Participation in France does not generate financial compensation for participants. This would suggest that for some patients, indirect costs [35] may be a barrier to being invited. This financial-based inequity and other inequities related to living in a rural area or being provided care in small local private care centers have recently been remedied through the organization and funding of *équipes mobiles* (mobile clinical research teams), the aim being to increase research in all care centers in the country and to help patients who agree to participate in clinical trials to reach reference centers by covering their transportation and housing costs.

Our study showed that the type of cancer care was strongly related with the invitation to participate in clinical trials. This is consistent with the international literature [36] and with previous French data showing that 39% of participants in a cancer-based trial was recruited in comprehensive cancer centers [34]. In addition, a French study on patients with stomach cancer showed that invitations to participate in clinical trials were less likely in private institutions that had fewer human resources dedicated to clinical research [37]. To remedy this, structural solutions must be put in place such that community and public academic centers can refer patients with treatment failure to comprehensive centers to ensure equitable access to clinical trials. Moreover, we found that patients living in a large town/city were more likely to have been invited to participate in a clinical trial. This is in line with findings from previous studies on geographical disparities in cancer clinical trial participation [38,39], and it could be partly explained by easier access to comprehensive centers that tend to be located in or near large towns/cities.

Efforts have also recently been made to increase the participation of older cancer patients in clinical trials. Our results confirm that this sub-population was less likely to have been invited to participate in a clinical trial, even after adjustment for major comorbidities. This would suggest that non-eligibility might not fully explain why older people are less likely to be invited. The 5.6% of patients over 65 years old in our study who were invited to/agreed to participate is consistent with previous estimates and with France’s national objective to increase participation to 5% in elderly patients [34]. Another possible explanation as to why older patients were less likely to be invited was that they might have been perceived as having a lower HL level. However, the age effect persisted after accounting for HL level. Accordingly, we can suppose that advanced age is a major factor in inequality in access to clinical trials. The hypothesis that subjective elements (e.g., compliance, attachment, comprehension, etc.) may explain this result cannot be dismissed.

After multiple adjustments, limited HL remained associated with a lower likelihood of being invited to participate in a clinical trial, highlighting that HL is a source of inequity for all cancer patients irrespective of age. This confirms our study hypothesis and the—albeit scarce—literature showing that low HL could be a barrier to participation in cancer clinical trials. Although health literacy was significantly associated with an invitation to participate, education level was not. This suggests that physicians are more sensitive to their patients’ level of understanding than to their level of education. One might argue that not suggesting participation to those perceived as having low HL might be something positive, if the research actors involved are not able to obtain full informed consent. This is particularly relevant in terms of oncology 3.0, where consent forms continue to increase in length and complexity. However, it seems more reasonable to consider limited HL as a social inequity that must be combated.

Although HL is associated with the invitation to participate in clinical trials, this does not rule out a possible literacy bias. Some patients might not be invited to participate in clinical trials independent of HL but in relation to other factors, including misunderstanding of clinical trials, socioeconomic [40], contextual, or other. Thus, our results should be interpreted with caution and should underline interest to consider, in the future, a holistic approach to improve patient’s understanding of clinical trials [14] in order to increase participation of all population categories. A lack of knowledge about clinical trials can influence trial participation (i.e., invitation and agreement) [41], and specific interventions have been proposed to improve this situation [42]. Such interventions include: (i) patient education to improve clinical trial knowledge, (ii) early distribution to the patient of a list of questions about the clinical trial in order to stimulate discussion with the physician when discussing possible participation [42,43], (iii) simplifying information documents to make them easier to read and understand for low HL patients [44,45], (iv) developing standardized patient decision aids to improve cancer trial recruitment [46], (v) and creating patient guides to help patients with limited HL to better understand clinical trials. Patient guides have already been shown to help overcome barriers to screening for cancer [47]. All things considered, we believe that multilevel interventions are likely to be the most effective method to increase participation [48].

Our study has limitations. We did not measure the reasons why patients agreed to participate in a clinical trial (e.g., to further research, new therapeutic offer, etc.) or the differences between the level of offer and demand. However, previous results have shown that older patients, for example, are interested in participating in research [49]. Moreover, HL was measured using a single item screening tool evaluating only functional HL [24], and we cannot exclude the possibility of a relationship whereby those invited to participate received complex consent documents and were therefore more likely to declare difficulties understanding hospital materials. However, such a relationship would only have led to an underestimation of the association between the invitation to participate in clinical trials and adequate HL. Finally, trial invitation was self-reported and might have been prone to recall bias.

## 5. Conclusions

Stronger efforts must be made to ensure that the invitation to participate is not exclusively targeted at those with a better socioeconomic position and greater HL, given that disadvantaged populations, often with limited HL, are more affected by cancer. Addressing HL is necessary to create a more inclusive health system and to reduce inequalities in access to innovative cancer care.

## Figures and Tables

**Table 1:** Main patient characteristics as a function of invitation to participate in a clinical trial (N = 1954).

		Overall Population	Invited to Participate in a Clinical Trial		
		%	No (89.7%)%	Yes (10.3%)%	*p* Value
Age at diagnosis (years)	≤65	77.2	76.1	87.5	<0.001
	>65	22.8	23.9	12.5	
Cancer management center	Private center	50.8	52.5	36.0	<0.001
	Comprehensive cancer center	10.5	8.7	27.0	
	Public academic or community center	31.7	31.6	32.5	
	Missing/unknown	7.0	7.3	4.5	
Gender	Man	35.9	37.4	23.0	<0.001
	Woman	65.1	62.6	77.0	
Education level	No diploma	6.6	6.7	5.5	0.214
	<upper secondary school certificate	43.0	43.5	38.2	
	≥upper secondary school certificate	50.4	49.8	56.3	
Health literacy	Limited	37.6	38.9	25.9	0.001
	Adequate	62.4	61.1	74.1	<
Area of residence	Rural/Small town/city (<200,000 inhabitants	65.9	67.0	56.5	0.005
	Large city (≥200,000 inhabitants)	32.9	31.8	43.0	
	Missing/unknown	1.2	1.2	0.5	
Deprivation index	Low (<Q1)	24.7	24.2	28.5	0.299
	Intermediate (Q1–Q3)	50.3	50.3	50.5	
	High (>Q3)	24.5	24.9	21.0	
	Missing/unknown	0.5	0.6		
Financial resources	Low (<Q1)	22.3	23.2	15.0	0.028
	Intermediate (Q1–Q3)	46.5	46.4	47.0	
	High (>Q3)	24.6	23.9	31.0	
	Missing/unknown	6.6	6.5	7.0	
Cancer type	Breast	43.4	41.9	56.2	<0.001
	Lung	3.6	3.2	7.5	
	Prostate	16.7	17.8	7.0	
	Upper aero-digestive tract	3.5	3.4	4.0	
	Bladder	2.9	3.3		
	Kidney	3.7	3.9	2.0	
	Thyroid	5.6	5.9	3.0	
	Non-Hodgkin lymphoma	3.3	3.1	5.0	
	Melanoma	4.8	4.7	5.5	
	Cervical	2.3	2.2	3.5	
	Endometrial	1.0	1.0	0.5	
	Colorectal	9.1	9.6	5.0	
Metastases at diagnosis	No	98.3	98.4	97.5	0.266
	Yes	1.7	1.6	2.5	
Individual chronic condition score	Mean (SD)	0.73 (0.38)	0.73 (0.38)	0.76 (0.36)	0.204

Q1: first quartile; Q3: third quartile.

**Table 2:** Variables associated with the invitation to participate in a clinical trial (binary logistic regression, N = 1954).

		Trial Invitation			
		Adjusted Odds Ratio	95% Confidence Interval		*p* Value
			Lower	Upper	
Health literacy level	Limited	0.55	0.39	0.77	0.001
	Adequate	1			
Age (years)	≤65	1			
	>65	0.60	0.37	0.97	0.039
Management Center	Private	1			
	Public academic or community center	1.43	0.99	2.06	0.055
	Comprehensive cancer center	3.62	2.37	5.54	<0.001
	Missing/unknown	0.82	0.39	1.69	0.594
Area of residence	Rural/Small town/city (<200,000 residents)	0.67	0.49	0.92	0.014
	Large city (≥200,000 residents)	1			
	Missing/unknown	0.51	0.08	3.39	0.490
Financial resources	Low (<Q1)	0.54	0.33	0.88	0.014
	Intermediate (Q1–Q3)	0.82	0.57	1.18	0.301
	High (>Q3)	1			
	Missing/unknown	1.03	0.53	2.02	0.918
Cancer type	Breast	1.82	0.90	3.66	0.095
	Lung	3.78	1.55	9.22	0.003
	Prostate	0.84	0.36	1.96	0.689
	Upper aero-digestive tract	2.16	0.82	5.64	0.117
	Bladder	0.09	0.00	4.56	0.232
	Kidney	0.94	0.28	3.18	0.921
	Thyroid	0.78	0.26	2.32	0.655
	Non-Hodgkin lymphoma	2.88	1.12	7.45	0.029
	Melanoma	1.86	0.73	4.75	0.195
	Cervical	2.60	0.90	7.47	0.076
	Endometrial	0.56	0.04	7.24	0.658
	Colorectal	1			
Individual chronic condition score, per one-point increase		1.31	0.86	2.00	0.212

## Data Availability

The data presented in this study are available on request from the corresponding author.

## Appendix A

English translation of the questions in the VICAN surveys regarding clinical trial invitation and participation.

A Clinical Trial is a scientific study evaluating a new drug not yet available or a new treatment approach. It is conducted in a hospital with volunteers who provide written signed consent to participate.

Have you ever been invited to participate in a Clinical Trial?

□ Yes    □ No    □ I don’t remember

If yes: Did you agree to participate?

□ Yes    □ No   □ I don’t remember
